# Evaluating intra-action reviews at points of entry: ongoing learning opportunities during the COVID-19 pandemic

**DOI:** 10.1186/s12889-022-14706-4

**Published:** 2023-01-06

**Authors:** Doret de Rooij, Miriam van de Watering, Remco van Dijk, Thijs Veenstra, Rolf Appels, Corien Swaan, Aura Timen

**Affiliations:** 1 Centre for Infectious Disease Control, National Institute for Public Health and the Environment, Bilthoven, The Netherlands; 2 Athena Institute, Free University of Amsterdam, Amsterdam, The Netherlands; 3 Faculty of Medical Sciences, Radboud University, Nijmegen, The Netherlands; 4 Department of Primary and Community Care, Radboud University Medical Centre, Nijmegen, The Netherlands

**Keywords:** Intra-action review, In-action review, Infectious diseases, COVID-19

## Abstract

**Background:**

Long-lasting crises, such as the COVID-19 pandemic, require proper interim evaluation in order to optimize response. The World Health Organization and the European Center for Disease Control have recently promoted the in(tra)-action review (IAR) method for this purpose. We systematically evaluated the added value of two IARs performed in the Dutch point of entry (PoE) setting.

**Methods:**

Two online, 4-hour IAR meetings were organized in March 2021, for ports and airports respectively, to reflect on the ongoing COVID-19 response. Topics discussed were selected through a survey among participants. Participants were mainly self-selected by the (air)port public health service. Evaluation of the IAR method consisted of participant evaluation through a questionnaire, and hot and cold debriefs of the organizing team. Evaluation of the impact of the IAR was done through analysis of the meeting results, and a 3-month follow-up of the actions proposed during the meetings.

**Results:**

Thirty-nine professionals joined the IAR meetings. In the participant evaluation (*n* = 18), 89% agreed or totally agreed the IAR made it possible to identify challenges and problems in the COVID-19 response at PoE. Participants especially appreciated the resulting insight in regional and national partners. Regarding the online setting of the meeting, participants suggested to choose accessible and familiar online tools. After 3 months, all national actions and actions for ports had been executed; some regional actions for airports required further attention. A major result was a new meeting structure for all ports and the participating national authorities in which remaining and newly occurring issues were discussed.

**Conclusions:**

Based on the evaluations, we conclude that the IAR method can be of value during long-term crises, such as the COVID-19 pandemic response. Although it is challenging to dedicate time and effort to the organization and attendance of IAR meetings during crisis, the IAR method is feasible in an online setting if appropriate organizing and technical capacity is available. A participatory set-up supports the IAR method as a starting point for continuous exchange and learning during ongoing crises.

**Supplementary Information:**

The online version contains supplementary material available at 10.1186/s12889-022-14706-4.

## Background

Prevention and control of internationally spreading infectious diseases are crucial in protecting public health. However, as stated in the International Health Regulations (IHR), “unnecessary interference with international travel and trade” should be avoided [1]. This international aim has implications for local operations. Although the interference in international travel and trade depends on decisions made on country level, it should be dealt with locally at points of entry (PoE) – airports, ports and ground-crossings. It is in these local settings that stakeholders represent different interests, all working towards both healthy, safe and ongoing travel and trade. Therefore, a tailor-made approach is desired in scarce but impactful crises such as pandemics [2], leading to a complex but crucial cooperation among stakeholders at PoE [3–6].

To support efficient responses at PoE, core capacity requirements for designated PoE are explicitly stated in the IHR. The status of the implementation of these capacities should be reported yearly in the Electronic State Parties Self-Assessment Tool (e-SPAR). Core capacity implementation for PoE has been one of the lowest scored indicators in the e-SPAR [7]. The transport and public health sectors have made extensive preparedness efforts on (inter)national and local level to prepare PoE to deal with outbreaks [8–12]. Still, the COVID-19 pandemic has shown that the response to a pandemic is challenging, can lead to severe interference in international travel and trade [13–17], and creates high demands for cross-sectoral collaboration.

Usually after a crisis, reflection and evaluation programs such as after action reviews (AAR) are performed. Based on AAR results adjustments in the preparedness plans and practice to further improve preparedness and response [18, 19]. However, waiting till after a crisis may be insufficient while dealing with challenges during a crisis, especially if the crisis is long-lasting. Both the World Health Organization (WHO) and the European Center for Disease Control (ECDC) have lately developed methods for in- or intra-action reviews (IAR) [20, 21]. The IAR aims to perform a quick evaluation and reflection during a crisis on national or subnational levels, bringing forth the lessons, best practices and most important needs to support the response that is ongoing. Serving the crisis situation with limited time and preparation, the IAR should be a fast and hands-on method that can be used for learning and improvement during a crisis.

The National Institute of Public Health & the Environment (RIVM), which is also designated as IHR National Focal Point for The Netherlands, conducted two IARs focused on the COVID-19 response; one for stakeholders involved at Dutch seaports and one for Dutch airports. As the IAR is a fairly new methodology, we evaluate the two IARs in the Netherlands, based on the research question: *How does the IAR methodology support enhanced, ongoing response to COVID-19 at POE?* To answer this question, we first describe the methodology of the IAR for this specific context. Secondly, we evaluate the impact of the IARs on the COVID-19 response through the IAR session results, recommendations and experiences from participants. Thirdly, we report on the follow up of actions in the response among participants after a 12-week period. We end the study with recommendations resulting from our experience on the IAR methodology.

## Methods

We conducted two IARs following five steps, based on the WHO and ECDC frameworks [20, 21]. The first step is the design of the meeting. Here discussion topics and participants were identified based on input via a questionnaire (design). Next, two 4-hour online meetings were prepared for partners at ports and airports respectively (prepare). During these meetings, best practices, lessons and needs were exchanged and actions for the near future were formulated (execute). Results were shared among the participants and national and international partners to exchange results and experiences with the IAR method (report). After the IAR the meetings were evaluated among participants, as were the recommended actions after 3 months (evaluate). An overview of these steps can be found in Table 1. In the subsequent section, we present the public health context in which these IARs took place, and elaborate on the methodology for each of the five steps.

**Table 1:** **Overview of the organization of the two IARs**

**Steps**	**Design**	**Prepare **	**Execute**	**Report**	**Evaluate**
**Timing since the start **	Week 1-4	Week 5-8	Week 8-9	Week 9-16	Week 9-16
**Working hours**	90	110	40	90	80
**Activities**	•Subject decision •Formation IAR team •Exploration of participatory methods •Questionnaire	•Collection of topics •Set meeting agenda •Preparing session materials	•Briefing •Online session •1^st^ Debrief & evaluation •Information management	•Finishing action plan •Report to participants and international partners	•Follow-up on actions •Evaluating the IAR organization
**Developed materials**	•IAR Project plan •Invitation and questionnaire on urgent topics and partners present	•Meeting script & agenda •Attendance sheet •Covid-19 timelines •Powerpoint presentation •Mural online cow sheets •Working sheets •Mentimeter questions	•Collaboratively designed plan in ppt slides •Meeting recording •Draft meeting minutes •Meeting registration •Used white boards of participants	•Draft meeting report •Final report •Short writing on IAR results for international partners •Summarizing video	•Final report •Participant feedback form + evaluating on content •Organizing team feedback form •Follow-up plan on actions feedback form

### Context of the IARs

These IARs were conducted by the WHO IHR National Focal Point (NFP) of the Netherlands (RIVM) [22]. In the context of the IHR, the Netherlands has two designated ‘A-PoE’: Amsterdam Airport Schiphol and the Port of Rotterdam. In addition, four other airports and 15 other sea ports process substantial international travel and therefore have a national status as ‘B-PoE’ [23]. At both ports and airports, the day-to-day infectious disease control is carried out by the regional Public Health Services (PHS). The legislative tasks of the PHS and PoE authorities are described in the Dutch Public Health Law [24], which incorporates the IHR requirements [1]. The PHS are supported by national guidelines developed by the RIVM. The RIVM also coordinates the public health response during severe communicable disease outbreaks, and can be consulted by the PHS. The PoE authorities and PHS collaborate with the Safety Region, responsible for preparedness and the management of crises, disasters and disruptions of public order [25]. The public health measures on a national level - as for COVID-19 - are imposed by the national government and implemented by the respective ministerial departments.

The IARs were conducted on 11 and 18 March 2021, during a period of widespread and increasing community transmission of COVID-19 in the Netherlands; the so-called “third COVID-19 wave” . Several public health safety measures were in place for the general population and at PoE in particular, such as mandatory self-quarantine and isolation, and mandatory testing before entering the country. Shops, bars, and restaurants were closed and a nightly curfew was in place. A flight ban for travelers coming from South Africa and several Latin American countries was in place, while a flight ban and a mooring ban for travelers coming from the United Kingdom was just lifted on March 9. Travelers coming from “high risk countries” were obligated to show both a negative PCR and a negative antigen test before entering the country [26].

### Design

We had to organize an online IAR meeting because of the public health measures in place at that time. The two meetings were organized via Webex Teams [27].

#### The organizing team

The team conducting the two IARs consisted of seven members, and technical and communication support. Members were all but two directly involved in the COVID-19 response in the Netherlands, including at Dutch PoE. Previous relevant experience of team members included: supporting and performing AARs, organizing and coordinating training and exercises in pandemic preparedness, and scientific experience in evaluating capacity building events.

#### Sampling

The selection of participants was partly done by the RIVM, but extended with participatory methods. The RIVM invited the PHS serving a PoE in their region to join the IAR. These PHS were asked to further recruit participants by inviting key partners in the COVID-19 response at their respective PoE through their own channels. This recruitment was facilitated by a standard invitation letter and the link to an online questionnaire (Formdesk [28]). This questionnaire contained an open question who should be present at the IAR meeting. The questionnaire for ports can be found in Additional file 1. Based on the input via the questionnaire, the RIVM suggested further invitations of local partners to the PHS. However, the final decision to invite these additional local partners remained up to the PHS. As the Ministry of Health (MoH) has a pivotal role in the COVID-19 response, the RIVM invited a representative as consultants during the IAR meetings.

#### Agenda setting

The questionnaire that was sent for recruitment also contained open and Likert scale questions on discussion topics for the IAR. Based on the results of the questionnaire, the potential topics were listed. Professionals from the RIVM who are involved in the COVID-19 response at PoE (TV and RA) sought common ground among all suggested topics. This resulted in two major topics to be discussed during the IAR meeting. The two major topics chosen were: A - implementation of various control measures, and B - cooperation: communication, tasks, and roles. For each of these two topics, several sub-topics were listed that could further steer the direction of the discussion during the meeting.

### Prepare

**Fig. 1:**
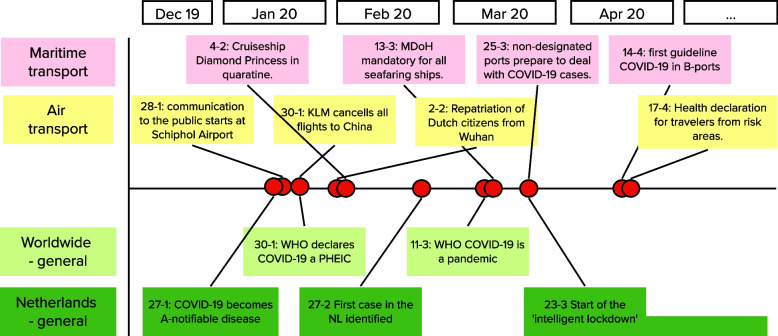
Timeline example with COVID-19 public health measures and developments relevant for Dutch PoE

As suggested in the WHO guide, “a structured review of activities undertaken at the national and subnational levels” was done. Two timelines – for ports and airports respectively - of events in the COVID-19 response were developed. Input for these timelines came from: a brain storming session with experts from the RIVM; data collection from a timeline in progress at the MoH; the national Outbreak Management Team advice letters to the government; and the governmental letters to the Dutch Parliament [26, 29, 30]. Figure 1 shows a part of the timeline with major events in the Netherlands and transport sectors relevant for Dutch PoE. A more extensive version of this timeline was used during the IAR and available to all participants. In addition to a presentation supporting the IAR meeting, materials to support understanding of the scope of the IAR for participants were developed. These included work sheets for the different breakout sessions during the IAR meeting, tools to gather input efficiently, relevant trigger questions [20, 21], and an online whiteboard (Mural [31]) to facilitate discussion and a root-cause analysis [20]. The preparatory materials and materials for the IAR breakout sessions during the IAR for ports can found in Additional file 2 and Additional file 3; these resemble those for airports.

### Execute

#### Meeting participants

The facilitating team consisted of a moderator, two technical assistants, two experts of the RIVM, and four moderators for subgroups who also acted as support during the meeting (note taking, solving issues for participants). During the IAR for sea ports, an observer with extensive experience with AARs and IHR implementation was present as well. PHS and authorities from all airports and all but one sea port were represented during the IAR meetings. PoE were represented by professionals affiliated at the PHS, the Safety Region and/or the airport or port authorities. The exact list represented PoE and participant affiliations is shown in Table 2. One week before the online meeting all participants had received documents for preparation. Participants had the opportunity to join a test session to prevent technical hick-ups during the session.

**Table 2:** Participants of the IAR meetings for airports and ports respectively

Airports		Ports	
Schiphol (designated); Rotterdam; Eindhoven; Maastricht-Aachen; Eelde Airport.		Rotterdam (designated), Moerdijk, Schiedam, Maassluis, Dordrecht, Vlaardingen; Amsterdam, Velsen/IJmuiden, Beverwijk, Zaandam; Vlissingen, Terneuzen; Delfzijl, Eemshaven; Harlingen.	
**Organization (number of participants)**	**Job title**	**Organization (number of participants)**	**Job title**
Public Health Service (*n* = 5)	Medical doctor Infectious Disease Control (2x); Medical doctor; Nurse Infectious Disease Control; project manager.	Public Health Service (*n* = 15)	Medical doctor Infectious Disease Control (4x); medical doctor (3x); Nurse Infectious Disease Control (2x); Inspector ship sanitation (4x); Infection prevention & control expert; Senior policy advisor.
Safety Region (*n* = 3)	Manager (2x); senior policy advisor.	Safety Region (*n* = 1)	Strategic advisor
Airport authority (*n* = 4)	Team lead Airport Operations Officers; Manager Security & Safety Operations; Airport Manager; Senior Policy Advisor.	Port authority (*n* = 5)	Coordinator shipping affairs; port security officer (2x); advisor port safety; advisor port health.
Government (*n* = 1)	Senior Policy Officer.	Government (*n* = 1)	Senior Policy Officer.
RIVM (*n* = 2)	Head of Prevention & Control Department; Medical doctor Infectious Disease Control.	RIVM (*n* = 2)	Head of Prevention & Control Department; Medical doctor Infectious Disease Control.

*RIVM* National Institute of Public Health and the Environment / WHO National Focal Point in the Netherlands

#### Meeting flow

Following an introductory round, the IAR methodology was explained, as well as the aim of the meeting, and the timeline of the COVID-19 relevant public health measures. Next, Topic A – implementation of public health measures – was introduced, with suggestions for subtopics and perspectives to trigger discussion. Participants ranked the subtopics through a poll in Mentimeter [32]. The most urgent subtopics (*n* = 3) were subsequently discussed in various break-out sessions. Break out groups consisted of participants from different regions, but we aimed at representation of each affiliation (e.g. PHS, safety region, port authority) in every group. The break-out room was introduced by the moderators but guided by participants themselves based on the pre-disseminated work sheets and the online whiteboard. To ensure that participants felt free to discuss their issues and concerns, the MoH and RIVM consultants did not actively participate in the break out session but could be consulted upon request. Back in the plenary session, one representative of each group presented the findings, after which there was room for additions, questions and discussion by all participants. The focus lay on formulations of concrete actions to be done by the different accountable partners in the short and middle-long term of the COVID-19 pandemic. After the break, the abovementioned method was repeated in similar order for Topic B – regional and supra-regional cooperation. Break-out groups now consisted of partners in the same and adjacent regions.

The online IAR meeting ended with a plenary summary and reflection session. Any remaining issues were discussed, and we provided the opportunity to share any remaining concerns, questions, or advice. To allow for thoughts to be shared, participants voted on statements such as: ‘I could share today what I planned to share’, ‘other urgent issues not discussed are…’. The results were directly discussed with participants.

### Reporting

A draft report was developed based on the notes taken during the online meeting as well as the digital whiteboards used in the break-out sessions. The aim was to summarize concrete lessons, best practices, challenges and actions formulated during the meeting. Where missing information was identified, e.g. an action had insufficiently be designated to a specific partner, a suggestion was made in the report for the missing information. The draft report was sent to all participants for their feedback and/or approval. After implementation of the feedback in line with the content of the IAR meeting, a final report was disseminated to the IAR participants. Our experience with the IAR methodology was shared with WHO EURO, three European countries interested in organizing an online IAR, the coordination of the EU HEALTHY GATEWAYS joint action, and in a training of the IAR methodology within the EU SHARP joint action.

### Evaluation

**Fig. 2:**
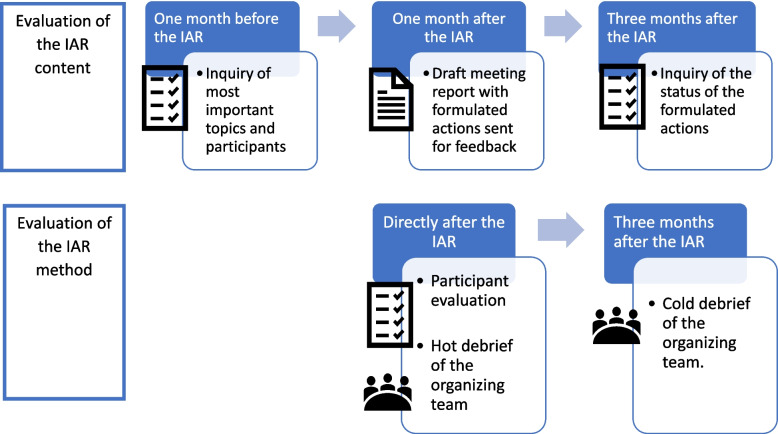
The evaluation steps of the IARs

The evaluation of the IAR was twofold, focusing on both the experience with the IAR methodology, and the impact of the IAR in practice through the monitoring of the implementation of formulated actions during the IAR meeting (Fig. 2).

#### Monitoring the implementation of actions

In the weeks after the IARs, efforts were made to monitor follow-up of the action plan that had been formulated during the IAR meetings. There was iterative contact between the participants and the RIVM on the follow-up of recommended actions. After three months an additional questionnaire was sent to all participants in which feedback per recommended action was collected (Additional file 4).

#### The IAR methodology

An evaluation of the IAR methodology was organized among both the organizing team and IAR participants. Participants received an online evaluation form directly after the IAR meeting (Additional file 5), followed by several reminders during the following weeks. The evaluation form was based on the example provided by WHO [20]. An open question on participants’ personal take home messages was added. The questionnaire was analyzed by calculating mean scores and standard deviations and comparing results for ports and airports using the Mann Whitney U test (non-parametric test comparing small groups), α < 0.05. The organizing team performed a hot debrief to discuss the most remarkable moments regarding method and content of the IAR meetings. Three months after the IAR, lessons learned were formulated collaboratively in a cold debrief. Subsequently, the IAR script was updated for any subsequent IARs.

## Results

In this section, we subsequently present the results from the IAR meeting with airports and the IAR meeting with the seaports. These include the results of the IAR meetings, the implementation of actions 3 months after the meetings, and the results of the participant evaluation questionnaire.

### Airports

The IAR meeting with the airport representatives (*n* = 15) produced a set of best practices, challenges and recommended actions. The best practices shared by the participants included short communication lines, mutual trust, and an informal and pragmatic ambiance among partners in the region. In addition, the accessibility of the MoH was named as a good practice by airport authorities, as was the centralized approach of public health investigations and contact tracing of flight contacts by the PHS serving Schiphol Airport. Topics of concern included the communication to traveler flows. Different countries of departure, countries of destination, in- and outside EU/EEA led to different risk profiles and subsequent communication needs. Recommended actions focused on timely communication from the national to the local level, and developing an enhanced overview of meeting structures on both national and regional level. A complete overview of discussed topics, recommendations, and results of the follow-up after 3 months can be found in Table 3**.**

**Table 3:** Output of the IAR meeting for airports

	Discussion topics	Recommended actions agreed upon during the IAR	Follow up after 3 months
**Session 1 - Public health measures**	• Difficulties to timely implement mandatory public health measures after announcement on a government level; • The implementation of measures practically varies per airport due to their size and specific setting; • The enforcement of behavioral regulations, such as social distancing, is difficult at airports; • Operations of monitoring the mandatory public health measures for travelers; • Unequivocal and clear public communication to incoming travelers.	• Involve local stakeholders timely in the drafting of measures on a national level to increase suitability and applicability to practice and involve the RIVM when needed; • Inquire whether tailor-made implementation of measures is possible for airports other than Schiphol; • It is strived for to include an infectious disease consultant in the 3-weekly meeting structure between the MoH and airport authorities. The RIVM is invited when deemed necessary; • The signal of the difficulty of maintaining the behavioral regulations is shared with the respective national security authority;	• An infectious disease consultant has been included into the 3-weekly meeting; • Signals on tailor-made implementations and the control of measures had been shared and discussed on a national level.
**Session 2 – Regional, supra-regional and national collaboration**	• Short communication lines, mutual trust, and an informal and pragmatic ambiance among partners in the region was considered essential and established in the majority of the regions; • An enormous expansion of staff at the PHS and high turnover in staff complicates cooperation among partners; • Discussion among partners from different regions as well as with the MoH and the RIVM is considered valuable; • Centralized contact-tracing for flight contacts by the PHS for the A-port is considered efficient and should be continued; • It is important to provide insight in the different meeting structures that exists between regions and on a national level;	• Regional partners keep investing in their regional network, now and in inter-pandemic times; • The regional PHS and airport authorities for each airport will map out the existing cooperation structures, and identify ways to optimise it; • A clear overview of the existing national platforms and meetings is provided by the MoH; • At Schiphol airport, an inventory for agreements will be done to deal with stranded transit passengers.	• Regions stressed that these issues require continuous attention, but no concrete regional actions were noted; • The MoH has provided an overview of existing national platforms; • At Schiphol, a protocol has been developed to deal with stranded travelers;

*PHS* public health service, *MoH* Ministry of Health, *RIVM* National Institute of Public Health

### Ports

Good practices listed during the IAR for ports (*n* = 24) included high mutual trust, good relations, short communication lines, and recognition of each other’s expertise among partners in the regions. Also, the PHS of the smaller sea ports stressed the value of the advice from partners of bigger sea ports. Participants agreed that the RIVM is well accessible for questions and for cooperation with the regional partners. As an example, the development of a guideline for restarting cruises was considered a fruitful cooperation between the RIVM and the respective PHS. Particular challenges were among others the COVID-19 notification procedure for docked ships, the variety in providers of COVID-19 tests, and the applicability of national measures in the regional sea ports; regional collaboration; and national collaboration. An overview of main discussion topics is shown in Table 4**.**

**Table 4:** Output of the IAR meeting for ports

	Discussion topics	Recommended actions agreed upon during the IAR	Follow-up after 3 months
Session 1 – Implementation of public health measures	• The MDoH is an important tool to respond to cases on incoming ships, and its use for ships docked in the port; • Timely information on test results of the crew is essential but challenging due to the wide variety of testing companies; • Privacy regulations should be respected during handling of completed MDoHs and annexes; • The responsibility for implementation of measures and applicable exceptions for seafarers leave unclarities.	• Agreements on a regional level should be made on how to receive the MDoH from docked ships; • The PHS arrange clarifications on COVID-19 notification for ships docked in the port; • The PHS internally evaluate the handling of health information and other personal data; and stress the importance of data handling in line with applicable laws and regulations; • All regional public health authorities should designate a key figure for sea port related issues in their organisation to warrant developments and communication.	• Most PHS report they have made regional agreements on the notification procedure for ships docked in the port. • The PHS have started to or completed bringing the handling of health information and other personal data under the attention of the shipping agencies. • All but one PHS have designated a key figure for sea port related issues.
Session 2 - Regional, supra-regional and national cooperation	• Conditions for contact among regional partners in the context of heavily growing and changing staff; • The existing and needed inter-regional and regional-to-national consultations and exchange of experiences; • The restart of the sea and inland cruises are a major dossier in the upcoming months.	• A 3-weekly meeting will be installed by the RIVM to discuss on and cooperate in clarification, elaboration and tuning of (new) public health measures and policy for ports.	• The 3-weekly meeting has been organized several times by the RIVM, inviting the MoH and IAR participants; • All but one PHS have joined the 3-weekly meeting; • Recommended actions formulated during the IAR meetings had been followed up during the 3-weekly meeting; • Five out of seven responding PHS report the contact with the RIVM has remained well or has improved.

*PHS* public health service, *MDoH* Maritime Declaration of Health, *MoH* Ministry of Health, *RIVM* National Institute of Public Health

The follow-up performed after 3 months resulted in a new three-weekly meeting structure. The composition of this meeting is similar to the IAR meeting, including representatives of the MoH, PHS, port authorities and the Safety Region. Focus of the meeting is the easy exchange of newly emerging challenges, and consultations among regions and national authorities. As a result, the above-mentioned challenges identified during the IAR session have been further discussed to the extent necessary. Examples of new challenges discussed during this meeting are implementation of the vaccination for seafarers and the restart of sea- and inland cruises. An overview of recommended actions and the results during follow-up can be found in Table 4**.**

### Evaluation of the IAR meeting by participants

The evaluation form was completed by 7/13 (54%) participants for airports and by 11/26 (42%) participants for ports. Results between participants for ports and airports did not differ significantly. Overall, 16/18 (89%) agreed or totally agreed that the IAR made it possible to identify challenges and problems in the COVID-19 response at PoE. 17/18 (94%) agreed or totally agreed that the IAR made it possible to share experiences and solutions in the COVID-19 response at PoE. 15/18 (83%) participants agreed or totally agreed that the IAR methodology is effective for achieving objective and concrete results, while 3/18 (17%) were neutral on this subject. Statement 8, on the efficiency of session A (implementation of different control measures), was scored lowest (10/18 (56%) agreed or totally agreed) together with statement 14 (7/18 (39%) were neutral, and 11/18 (61%) agreed or totally agreed). Results of the questionnaire can be found in Table 5. The data where behind these results are provided in Additional file 6.

**Table 5:** Results of the evaluation questionnaire for participants of the IAR meetings

Evaluation questions	AIRPORTS (Mean ^**a**^(SD))	PORTS (Mean^**a**^ (SD))	Airports vs. ports^**b**^
1. The IAR made it possible to identify challenges and problems in the COVID-19 response in ports/on airports	4.29 (.49)	4.09 (.70)	*p* = .62
2. The IAR made it possible for participants to share experiences and solutions in the COVID-19 response in ports/on airports	4.00 (.58)	4.36 (.50)	*p* = .28
3. The IAR contributed to an improved cooperation amongst different public health partners and disciplines involved in the COVID-19 response in ports/on airports	4.14 (.69)	3.72 (.90)	*p* = .37
4. The IAR contributed to an improved multisectoral cooperation and coordination amongst involved parties in the COVID-19 response in ports/on airports	4.00 (.58)	3.63 (.81)	*p* = .39
5. During the IAR, there was room for participants to do suggestions how to improve the COVID-19 response in ports/on airports	4.29 (.49)	4.36 (.50)	*p* = .79
6. The presentation given on the method and process of the IAR meeting was clear and useful	3.86 (.69)	4.00 (.63)	*p* = .68
7. The introduction on the Dutch situation and timeline of important events presented were useful and efficient	4.29 (.49)	4.18 (.60)	*p* = .79
8. The first session, in which we discussed the implementation of different control measures in ports/on airports, went efficiently	3.57 (.98)	3.45 (.69)	*p* = .79
9. The second session, in which we discussed the cooperation in the COVID-19 control in ports/on airports, went efficiently	4.00 (.58)	3.91 (.54)	*p* = .79
10. The number of participants involved in the IAR meeting and its different sessions was adequate	4.14 (.38)	3.82 (.40)	*p* = .30
11. The participants involved in the IAR had the right profile to participate	4.14 (.69)	3.91 (.83)	*p* = .65
12. The methods used during this IAR could also be effective for evaluations of other subjects or events	3.86 (.69)	3.82 (.60)	*p* = .93
13. Generally, I consider the IAR methodology effective for achieving objective and concrete results	3.86 (.69)	4.09 (.54)	*p* = .50
14. The results of the IAR can contribute to put the most important defects in the COVID-19 response in ports/on airports timely on the agenda	3.86 (.69)	3.64 (.67)	*p* = .53
15. The results of the IAR can contribute to put the defects in coordination and cooperation on the agenda	3.86 (.69)	3.82 (.75)	*p* = .89
16. The results of the IAR can contribute to identify, repeat and retain solutions and efficient examples from practice	3.86 (.69)	4.00 (.63)	*p* = .68
17. The results of the IAR can contribute to support and strengthen individuals to improve handling the challenges of the COVID-19 response	3.43 (.79)	3.82 (.60)	*p* = .39
18. The results of the IAR can contribute to draw attention to solutions of new capacity developed during the COVID-19 response	3.86 (.69)	4.18 (.75)	*p* = .39

^a^Totally disagree = 1, disagree = 2, neutral = 3, agree = 4, totally agree = 5; ^b^P-value following from analysis via Mann Whitney U test

Feedback through the open questions resulted in a general positive stance on the IAR session and a better insight in regional and national networks. The IAR session is perceived as a valuable step in enhancing the network and the response. Participants described difficulties regarding the use of some of the digital tools used during the IAR. Unfamiliarity with the online environment, especially during the break-out sessions, partly drew away the attention from the point of discussion. It was advised to use well-known digital tools in future sessions.

## Discussion

We designed, executed and evaluated two online IARs among professionals involved in the COVID-19 response at Dutch ports and airports. In addition to the existing ECDC and WHO guidelines on IAR methods, we developed an inventorying questionnaire during the design of the IAR. This questionnaire facilitated the input of participants, prior to the actual conduction of the IAR, regarding the most urgent topics from their point of view and suggestions for inclusion of partners in the field. In this way, we could prioritize topics before the meeting and we aimed to create support for the IAR meeting and its follow-up. The IARs identified several good practices and challenges for airport and ports. Among others, the continuity of collaboration among partners involved at PoE showed challenging due to fast growing staff numbers and changing organization structures. Other challenges include the tailor-made and timely implementation of measures amidst a continuously varying pandemic situation. A major result of the IAR for sea ports is the new meeting structure for involved partners in the response. Based on the evaluation, we can conclude that the two 4-hour IARs with PoE proved feasible and valuable in the Netherlands, despite the extremely busy crisis context. We learned after a three-month follow-up that the exchange of experiences on an inter-regional and national level had continued beyond the IAR setting.

The evaluation of the IAR method produced similar results for ports and airports, with high satisfaction among participants and organizers. However, we observed better implemented actions in the port compared to the airport setting after 3 months follow-up. These differences may point out towards contextual factors outside this method that may play a role in the success of an IAR. In our study, the major difference in the results for airports and ports was the implementation of regional actions that followed from the IAR meetings. These actions were more often completed for ports than for airports. The direct consecutive meetings that took place for the ports may have attributed to this fact. During these meetings there was opportunity to further discuss and exchange about emerging situations. Also, the IAR results have been brought to the agenda several times by the RIVM that initiated the IARs and hosted these subsequent meetings. To the contrary, there was no new meeting structure introduced for the airport sector as a similar structure existed already between the MoH and PHS.

The discussion topics and recommended actions resulting from the IAR meetings served as the learning agenda for Dutch PoE in March 2021. Although specific needs and lessons may have changed at the moment of writing as the COVID-19 pandemic still continues, the IAR results can be of interest for PoE and national authorities in other countries. The specific challenges that one faces while trying to change practice often point towards more structural hurdles in the response. For example, the difficulty for non-designated PoE to implement measures in a tailor-made way during the COVID-19 pandemic may point out that structural attention is needed for these PoE and their role in this and future crises. Our suggestion to draw broader lessons of these results are supported by a recent study at German ports and airports, where similar important challenges are described. These include the dealing with the speed of new measures with hardly time to implement these in the specific airport’s setting, and the control of compliance with measures [33].

Literature on in action learning or the IAR method in particular is very scarce (literature search in May 2021). The WHO and ECDC guidance documents on how to apply this method are leading [20, 21]. The lack of a body of knowledge on this method is understandable as IAR is a rather new method. Also, performing IARs and learning in practice rather than describing them in literature might be deemed more important during the ongoing crisis at the moment of writing. We know from WHO communication and informal communications that several European countries have been conducting IARs in the last year. Therefore, we expect and warmly invite an expanding body of knowledge on this new methodology to emerge soon.

### Reflections on the methodology

Although we based the methodology of the IARs performed on the guidance documents from ECDC and WHO [20, 21], we state our recommendations and reflect on our choices in the application of the method in the Dutch PoE setting.

#### Online setting

The WHO guidance document advised to execute an IAR in an online setting [20]. However, online group meetings in general complicate bilateral informal exchange and require good technical conditions to function. A round of informal acquaintance at the start of the IAR meetings, called ‘ice-breaker’, was helpful to stimulate active participation. Another stimulator of active participation in the discussion that we experienced were small-group discussions led by a break-out session guide. The online whiteboard could visualize the group discussion, but was also considered time-consuming and unfamiliar by some participants. Details such as a common snack package that was sent to all participants can further simulate characteristics of offline meetings and create an ambiance of reflection and exchange. In general, the online working format should not be technically challenging for the participants to prevent difficulties and loss of time.

#### Choosing focus for the IAR

In line with the WHO guideline, we chose one of the suggested pillars and included partners that are active in the response at PoE. It became clear in the evaluation that meeting others in the network had been a valuable aspect of the IARs. Subsequently, we suggest to focus not only on choosing a pillar, but also to search for areas requiring new relations and with new interdependencies among parties compared to inter-pandemic times.

We integrated input of the IAR participants during the design, execution, evaluation and reporting of these IARs. We considered participatory methods valuable during the organization of these IAR, as we could focus easier during the meetings, and believe that the results mainly matter if they are corresponding to participants experiences. Previous literature shows that participatory studies and projects in general have larger impact if they are organized by people that are the end users of the results [34]. We emphasize that efficient impact is essential when focusing on learning during crisis times.

#### Follow-up

Participatory methods may also be helpful in the follow-up. Both ECDC and WHO guidelines name the essence of close monitoring of follow-up dedicated to a specific person or authority. However, there is no practical guidance on how to perform this follow-up. A more step-wise approach may be helpful and should at least include considerations to what extent follow-up on recommended actions should be monitored and by whom. Especially during crisis, burden of follow-up questionnaires or forms should be minimized. Therefore, the authors of this study consider that the better the design of the IAR has been performed in a participatory manner, the better the ownership of the results by the participants will be. This potentially will lead to a better follow up of recommended actions. Creating a network of partners around an IAR can support emergent follow-up and exchange, instead of a top-down monitoring process.

A thorough follow up after the IAR meeting would also better align with the plan-do-check-act cycle, a seminal theory of ongoing learning first introduced by William Deming. Following this approach, the implementation of recommended actions should be monitored and lead to new rounds of review whenever required. This is in line with WHO recommendations to organize series of IARs [20].

### Safeguarding capacity for learning during crisis

It was a challenge to combine the organization of the IAR with other professional duties. On average, seven people have spent two full-time working weeks with planning, preparing, performing, evaluating, reporting and follow-up of the two IARs presented in this paper. This team consisted of professionals with relevant experience to set up such a reflecting setting. The amount of time and effort invested by the team were much more substantial than suggested in the WHO guide, where there is a 1 week schedule for preparations before performing the IAR [20]. ECDC suggests brainstorming sessions that are less structured and in this sense may require less preparing time. We conclude that following these existing guidance was very helpful, but requires more preparations than described if a thoroughly prepared meeting is aimed for. It takes time to get familiar with the possibilities and choices and to develop the method in a way suiting the specific setting.

Despite the significant organizational burden of organizing an IAR, we highly recommend to perform an IAR. First, in our experience, the COVID-19 pandemic with its unprecedented scale and impact has led to new networks of cooperation in the response. For example Never before had Dutch ports and airports been in such a situation. However, the introduction of these networks is one of many challenges that have to be solved. The IAR creates a setting to get to know the other partners, see a face, and hear their story, to develop more sense of about what is happening in other parts of the response. Second, in a long-lasting crisis such as COVID-19, time for reflection must be scheduled as delaying it until after the crisis will downgrade the relevance of current lessons and needs. Both participants and our team confirmed that it was worth the effort despite all challenges. Finally, investments made in organizing an IAR for the first time may pay off in any future IAR. Shared experienced of other IARs may be helpful in providing a wide array of effort and times connected to outcome of this particular method. Sufficient capacity for in-crisis evaluation should be anticipated upon during preparedness planning.

### Strengths and limitations

We conducted one of the first IAR on COVID-19 in Europe. We have seen national, political and international attention for this method. This study is one of the first studies systematically evaluating an IAR. We based our method on existing frameworks from WHO and ECDC and experimented with the method in a specific setting (PoE). We thoroughly evaluated the IAR on several levels: feedback of participants, implemented actions, and efforts and impacts from the organizational team. This is in line with standards provided for evaluation of capacity building in cross-border settings [35].

We also see some limitations of this study of which we discuss the most important ones. First, the evaluation of the IARs may have suffered from response bias during evaluations in a sense that participants enthusiastic about improving the response provide often better feedback than people that may be less satisfied or indifferent regarding changes in practice. We tried to prevent this bias by performing several evaluations, reminding all participants several times, and actively performing a follow-up evaluation of recommended actions for all participants. However, only half of the participants have responded to the evaluation questionnaire. Second, the success of this IAR may have been supported by the evaluation of the IAR. Evaluating the IAR meeting and stated actions among participants is important to evaluate the method. However, it also brings the IAR and recommended action several times to the attention of participants, potentially stimulating further action. Lastly, we promote the integration of participatory methods, but the organizing team of these IARs has been solely from the RIVM. In this way, we have not entirely practiced as we preached. Especially in the selection of national authorities for the meetings, we have insisted on ‘our’ perspective of what would result in a fruitful meeting. We considered hierarchical relations, familiarity with suggested partners, group size and topics on the agenda. However, reflecting from our experience and the existing body of literature, it is clear that collaboration among partners in the IAR during the set up and organization leads to shared responsibility, better participation, and relevant impact in practice.

## Conclusion

This is to our knowledge the first study reporting of a systematical evaluation of the IAR method. We based our method on WHO and ECDC guidelines and applied it in an intersectoral sample of professionals involved in the COVID-19 response at Dutch airports and ports. According to participants’ and organizers’ views and in line with the results in practice we conclude that the IAR method can be of added value during a crisis. We highly recommend other countries to organize IARs during the, at the time of writing, current COVID-19 pandemic or any next long-lasting crisis. Especially the participatory approach and thorough follow-up should be considered as additional steps in this method. We furthermore stress the need to share, when appropriate, specific designs and the results of their design. In this way, we can collaboratively give further shape and substance to this very promising method.

## Supplementary Information

**Additional file 1.**

**Additional file 2.**

**Additional file 3.**

**Additional file 4.**

**Additional file 5.**

**Additional file 6.**

## Data Availability

The datasets generated and/or analyzed during the current study are only publicly available due to privacy and confidentiality reasons during the IAR process. Unpublished datasets are available from the corresponding author on reasonable request.

## Abbreviations

AAR: After action review
ECDC: European Center for Disease Control
e-SPAR: electronic State Parties Self-Assessment Tool
IAR: In- or intra-action review
IHR: International Health Regulations
MDoH: Maritime Declaration of Health
MoH: Ministry of Health
PHS: Public Health Service
PoE: Point(s) of Entry
RIVM: National Institute for Public Health and the Environment, Netherlands
WHO: World Health Organization
