# Supplementary material for: Evaluating intra-action reviews at points of entry: ongoing learning opportunities during the COVID-19 pandemic
Source: BMC Public Health. 2023 Jan 6;23:36. doi: 10.1186/s12889-022-14706-4 (PMC9816518; doi:10.1186/s12889-022-14706-4)
Supplement: Supplementary file 1 — Additional file 1. [file 12889_2022_14706_MOESM1_ESM.pdf]

# Additional file 1 – Inventorying Questionnaire (ports). Pdf file.

## Questionnaire as sent to participants for the IAR for ports

*[the original questionnaire was sent in Dutch]*

Dear colleague,

You are being invited to participate in an intermediate evaluation of COVID-19 response in seaports in The Netherlands. This evaluation meeting is scheduled on march 18, 2021 from 9:00 until 13:00 CET.

**In this questionnaire:**

- you can register for participation,
- we are asking your input for topics relevant for discussion in the evaluation sessions,
- and which parties you would like to participate in these sessions.

**Goal**

Together with the most important parties involved in the COVID-19 response in sea ports in The Netherlands, we would like to identify needs and lessons learned during the response so far, and to share and use these findings in the continuing COVID-19-respons. The goals is to supply both you and us with useful practical advice to enhance the COVID-19 response in sea ports.

**Participants**

To be able to effectively discuss challenges at hand, we aim for a diverse composition of the group of participants. We invite representatives of public health regions that serve an area containing an A- of B- port and ask them to select other parties and representatives that are involved in COVID-19 response in ports. You may therefor have been invited by LCI directly, or via the regional public health service.

**How is the evaluation performed**

In a later stage we will inform you about the program details. In short: we will follow the format of an 'in-action review'. This is a way of evaluating during crisis response that takes limited time. For these IAR standards are provided by ECDC and WHO.

**Would you like to participate?**

If you would like to participate, please register using this questionnaire and reserve the date of March 18th in your schedule. Early February 2021 an outlook invitation for the online session will follow, including technical details. As this is an online meeting (Webex), availability of a PC or laptop with video and a stable digital connection are required.

If you do not wish to participate because you estimate not to be the right representative for this subject, please suggest another participant to us. If there are any other reasons for you not to participate, we would kindly like to hear these reasons.

For any questions or clarification please reach us via lci@rivm.nl. We are looking forward to your reaction.

Yours sincerely,

Doret de Rooij, Miriam van de Watering, Rolf Appels, Thijs Veenstra and Corien Swaan

NB: Data you enter in this questionnaire will solely be used for the organization of the evaluation session, and for no other purpose. In any registration or documentation of the evaluation session, none of the participants response will be linkable to a specific participant. For any more information visit www.rivm.nl/privacy.

Please tick this box for giving consent:

1. From who did you receive this invitation?
   a. Direct from LCI/RIVM
   b. From a connection at the regional public health service
   c. Different, namely:

2. What is your name?

3. What is your profession?

4. What is your affiliation?
   a. Public Health Service
   b. Safety region
   c. Port
   d. Different, namely:

5. What is the name, including region (when applicable) of your affiliation?

6. Will you participate in the online evaluation (in-action review) on March 18 2020?
   a. Yes, I will participate and hereby I register;
   b. No, I will not participate

7. In which ways have you been involved in COVID-19 response in sea ports in The Netherlands?

8. Which three subjects seem most important for discussion during the meeting to you, as they are of hindrance in an effective and efficient COVID-19 response in ports?

9. To which extend seem the following aspects relevant to you??

| | **Not at all relevant** | **Not so relevant** | **neutral** | **relevant** | **very relevant** | **I do not know** |
|---|---|---|---|---|---|---|

| | | | | | | |
|---|---|---|---|---|---|---|
| Implementation of nationally imposed measures (general) | | | | | | |
| Use of the Maritime Declaration Of Health | | | | | | |
| Cooperation between Dutch regional public health services, ports, and other parties | | | | | | |
| Cooperation between national parties and parties in and around the port | | | | | | |
| Reacting on COVID-19-situations in ports and aboard ships | | | | | | |

10. If desired, clarify choices made above or add new subjects.

11. According to you, which parties are the most critical to participate in an in-action-review? Indicate if these are specific individuals or representatives of organizations.

12. When would the in-action review, being an intermediate evaluation, be successful in your opinion?

13. Do you have any other advice ore remarks in the approach of the in-action review?

This is the end of the questionnaire. U can submit your response by clicking the button below.

‘Send’
