# Supplementary material for: Evaluating intra-action reviews at points of entry: ongoing learning opportunities during the COVID-19 pandemic
Source: BMC Public Health. 2023 Jan 6;23:36. doi: 10.1186/s12889-022-14706-4 (PMC9816518; doi:10.1186/s12889-022-14706-4)
Supplement: Supplementary file 2 — Additional file 2. [file 12889_2022_14706_MOESM2_ESM.pdf]

Additional file 2 – Confirmation, Preparation and meeting materials (ports). PDF file. *[The original documents were sent in Dutch]*

Confirmation letter

Dear Colleague,

We hereby confirm your application for the In-Action Review (IAR, IAR) of the COVID-19 response at ports in the Netherlands. Below you can read the necessary information about the meeting. Thank you for your registration!

When and where: The meeting is scheduled for March 11, 2021 from 8:45 a.m. to 1:00 p.m. and will take place online via Webex Meeting.

Purpose: Together with key stakeholders in the COVID-19 response at ports we will discuss, share, and use the needs and lessons learned from the COVID-19 response, to use them in the continuation of the COVID-19 response. The goal is to learn from other, and to arrive at concrete advice and actions that need to be done to improve COVID-19 response at ports.

Practical Information: During the meeting we will use online tools (Webex, break out rooms, Mural) to make the evaluation interactive. There will be plenty of technical support prior to and during the sufficient technical support to help you with these online tools.

Optional: testing Webex: Webex works through an online link, you do not need to download a program or app for it. If you are not familiar with Webex and would like to test it (connecting, use, etc.), there is an opportunity to do so on Thursday morning, March 4 at 9.30 am. You can send an email to [e-mail address] We will then send you further instructions. Please indicate no later than Tuesday, March 2, if you want to participate in this test moment.

Permission for recording meeting and sharing results: We would like to make a recording of the meeting, for which we ask your permission. The recording will only be used for reporting the IAR meeting and will be destroyed afterwards. The results (not the recording) of the IAR meeting will be shared with other EU member states through a research report on the IAR. To agree to the recording and sharing of the results, we ask you to send an email with your agreement to the LCI (lci@rivm.nl). In this email you indicate your consent to the recording of the IAR meeting and the sharing of the IAR outcomes. This can be done by replying to this email.

Furthermore, one week before the start of the IAR meeting you will receive additional information, such as the content of the program, the list of participants and the login details for the Webex meeting. As a thank you for your participation and as a reminder of the meeting, you will receive a small gift. This will be sent to your organization's address in early March.

For any questions or clarification, we can be reached at lci@rivm.nl. We are looking forward to seeing you on March 11!

Preparation letter + meeting materials

Dear participant,

The goal of the In(tra) Action Review (IAR) on 18 March

The purpose of the In-Action Review (IAR) on March 18 is to reflect on corona control at ports based on your input. During the IAR, we will work solution- and future-oriented through an interactive program. What went well? What can be improved? And how will we tackle this for the future? We map out the most urgent challenges, try to clarify the underlying causes (root-cause analysis) and formulate concrete actions to prevent challenges in the future.

Preparation for an effective meeting

In order to make the best use of the IAR's valuable time, we ask the following in preparation:

For Session 1

- ➢ Explore the timeline of mitigation measures and events at the ports via: [link to the timeline].
- ➢ Are you still missing any key moments on the timeline that were relevant to corona response on ports?
- ➢ Please start thinking about the measures that had the greatest impact, caused the greatest problems or for which there are still the greatest gains to be made in the future. We will work with both the current challenges and/or the challenges that we foresee in the near future.
- ➢ How do these challenges relate to the available roadmaps, guidelines, and protocols you used during the corona response? Bring the relevant documents to the IAR. For your information: https://lci.rivm.nl/draaiboeken/international-health-regulations-ihr

For Session 2

- ➢ In preparation, consider concrete (practical) examples of collaboration in corona response. Both regionally and supra-regionally; and both where it went well and where it went not so well.
- ➢ Also pay attention in advance to why this collaboration is or is not going well. What is the underlying cause? What is needed to improve it?
- ➢ You will be encouraged to bring in actual case scenarios for illustration.

What you need for the IAR meeting

- A laptop or desktop (not a smartphone/tablet) with a working camera and microphone. Please note: WebEx does not work in a secure work environment such as Citrix, so make sure you work in the home environment.
- One day before the IAR (Wednesday, March 17) we will send you another document via email, make sure you can open it during the IAR.
- The WebEx login information, see below.
- The First-Eat kit (not necessary, has been sent to your work address).

We look forward to meet you on 18 March!

Part 1: Implementation of control measures in the port

Duration: 75 minutes

A. Plenary: Introduction (5 minutes)

Based on the timeline, we look at what control measures and situations in the COVID-19 control in the port had a major impact. We focus on those measures that caused the most problems and are still relevant today. We will look for the roots of the causes that caused these problems to arise. We conclude the session with concrete actions that will prevent or solve these problems in the near future.

B. Plenary: Problem identification (15 minutes)

Based on your previous input, we made a pre-selection of control measures whose implementation can be discussed in this session. You can vote in the Mentimeter for the specific measure you wish to discuss.

C. Break-outroom: Root-Cause analysis and formulating actions (25 minutes)

The group will be divided into 4 break-out rooms. Each break-out room will address one of the measures selected in the previous section. Please assign a chairperson and a timekeeper in your own break-out room.

Each breakout room has an online whiteboard (Mural) available for their support. The link to the whiteboard will be shared by the moderator. Use the following questions to analyze the problem at hand:

- How would you formulate the concrete problem?
- What went well during the implementation of the control measure at hand, and how come?
- What were challenges or problems encountered during the implementation of the measures?
    - How could these challenges / problems arise?
    - What is the cause of the cause of the problem? Try to reason towards the root of the problem.

Think about what might be a concrete solution or area for improvement. How could this be prevented from causing implementation problems in the future? As a group, appoint 1 spokesperson who will present in plenary afterwards.

D. Break (5 minutes)

E. Plenary: Exchange solutions (25 minutes)

Each group's spokesperson presents the findings in 5 minutes using the following 3 questions:

- What is the problem? What causes the problem? What are first suggestions for recommendations for the future?

After the presentations of all groups, the plenary moderator will summarize the recommended action points. In the plenary discussion that follows, recommendations will be adjusted and added.

Part 2: Cooperation: communication, tasks and roles

Break-out session

A. **Regional cooperation - duration: 15-20 minutes**

Please open the Mural – 'regional cooperation', the link will be provided by the moderator in the break out session.

In de Mural, you will find a table and a simplified overview of the different partners in the regional response. You will also find a table with questions guiding the discussion. The overview of partners and the table can be used to summarize and/or visualize the discussion.

The table:

1. Concrete examples of excellent regional cooperation	2. Concrete examples of sub-optimal regional cooperation
What are the conditions for this excellent regional cooperation?	What are the barriers towards optimal cooperation?
How are these conditions for excellent cooperation safeguarded in and among organizations?	How can these barriers be tackled in and among organizations?

Use practical examples if possible.

- Discuss at least one example of excellent cooperation in the region.
- Discuss 1-3 examples of suboptimal cooperation. What led to the suboptimal cooperation? What could be a concrete action to prevent this in the future?

Summarize:

- How cooperation goes well in the region and wat facilitates this cooperation.
- Concrete points for improvement regarding the cooperation in the region.
- A remaining question or problem regarding regional coopeation that could be presented to other regions.

Tip: Write down the summary and share it in the chat in the plenary session. Do not forget to mention what break-out session this input comes from.

B. **Supra-regional cooperation - duration: 15-20 minutes**

Repeat the exercise for the supra-regional cooperation. Supra-regional cooperation means cooperation among regions or with national authorities.

1. Concrete examples of excellent supra-regional cooperation	2. Concrete examples of sub-optimal supra-regional cooperation
What are the conditions for this excellent supra-regional cooperation?	What are the barriers towards optimal supra-regional cooperation?
How are these conditions for excellent cooperation safeguarded in and among regions?	How can these barriers be tackled in and among regions?

Summarize:

Supra-regional cooperation – input [region]

- To what extent the supra-regional cooperation has been considered effective.
- What are the needs to further improve supra-regional cooperation during the COVID-19 response, and why.
- One or more suggestions to improve supra-regional cooperation.

Tip: Write down the summary and share it in the chat in the plenary session. Do not forget to mention what break-out session this input comes from.

C. **Prepare feedback to the plenary** (3 minutes)

You will return to the plenary soon. Every group has the opportunity to present their findings. Please use the above-mentioned summaries, and add any other important findings.

D. **Plenary: exchange among regions** (30 minutes)

- Every region / group reports its findings via the plenary chat..
- We will discuss the similarity and recognition among regions for the different findings.
- We will collectively formulate recommendations and next steps.
