# Supplementary material for: Evaluating intra-action reviews at points of entry: ongoing learning opportunities during the COVID-19 pandemic
Source: BMC Public Health. 2023 Jan 6;23:36. doi: 10.1186/s12889-022-14706-4 (PMC9816518; doi:10.1186/s12889-022-14706-4)

Additional file 3 – Break out session materials (ports). PDF file. *[original documents were in Dutch]*

IAR ports – Blanco white board session 1

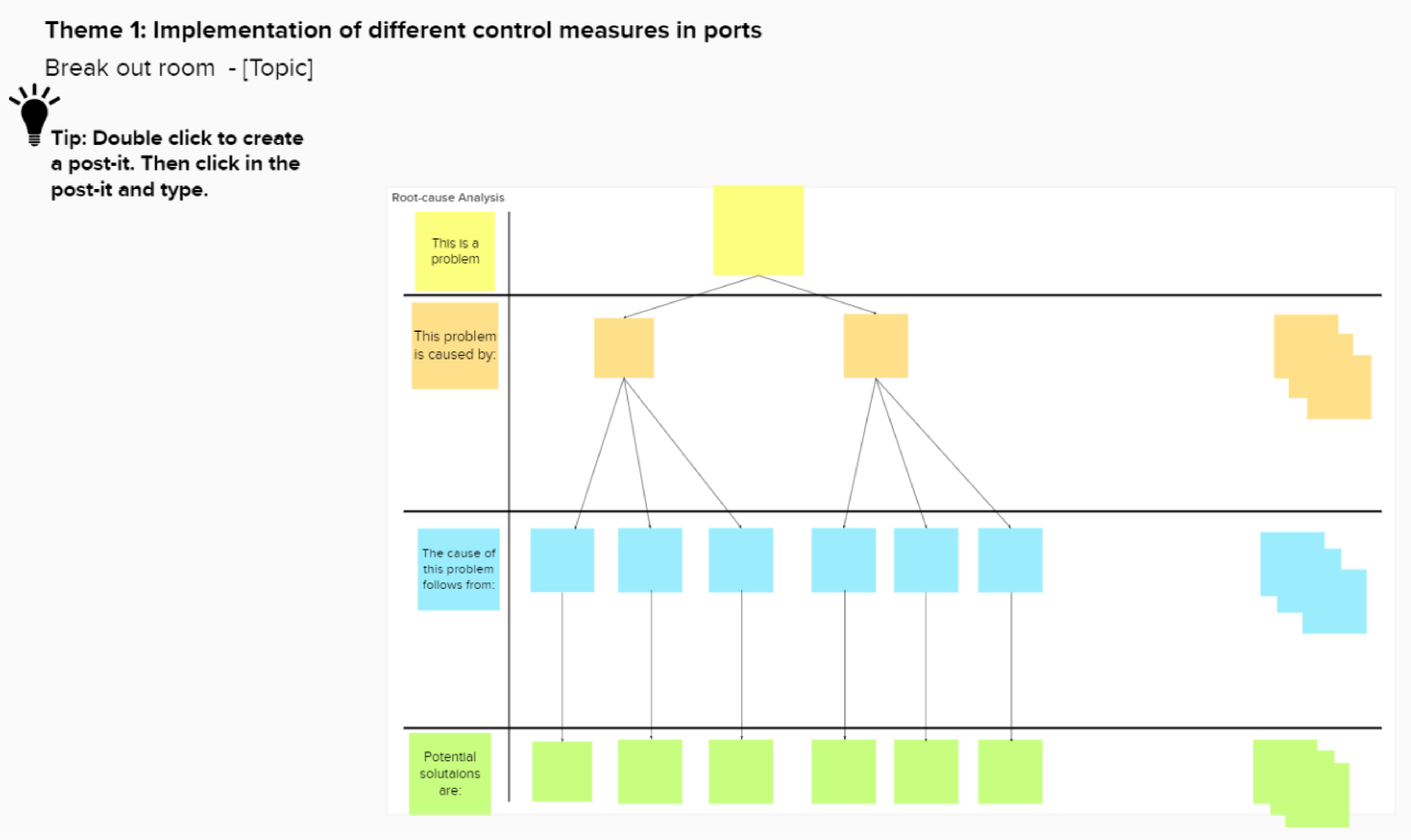

IAR ports – Blanco white board session 2

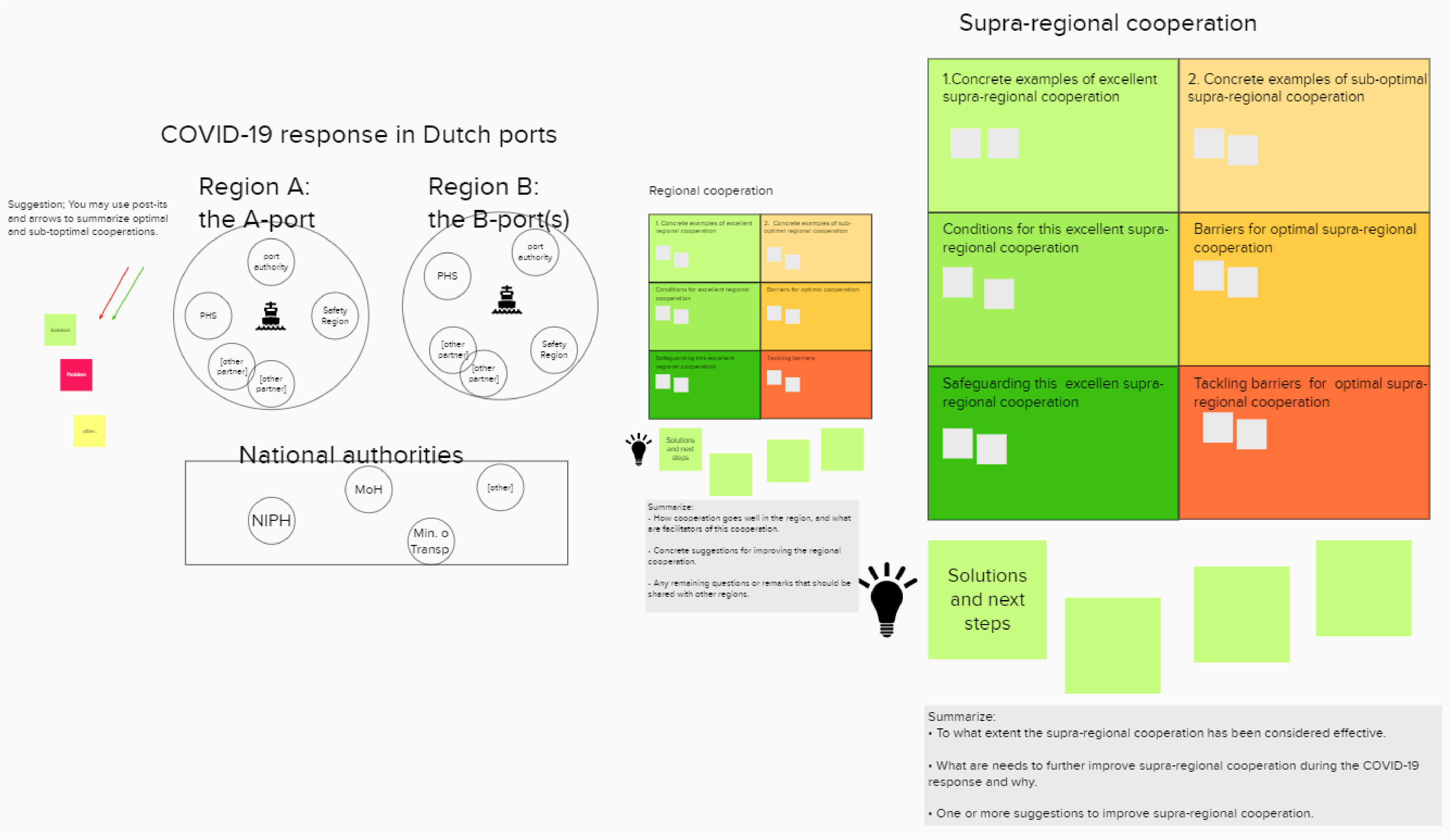

Supplement: Supplementary file 3 — Additional file 3. [file 12889_2022_14706_MOESM3_ESM.pdf]
