# Supplementary material for: Evaluating intra-action reviews at points of entry: ongoing learning opportunities during the COVID-19 pandemic
Source: BMC Public Health. 2023 Jan 6;23:36. doi: 10.1186/s12889-022-14706-4 (PMC9816518; doi:10.1186/s12889-022-14706-4)
Supplement: Supplementary file 4 — Additional file 4. [file 12889_2022_14706_MOESM4_ESM.pdf]

Additional file 4 – Participant evaluation form. PDF file. *[documents were sent in Dutch]*

COVID-19 IN(TRA)-ACTION REVIEW (IAR) (Air) Ports - Participant evaluation

1. **To what extent do you feel the following goals were achieved at the March 11 / 18 in(tra)-action review (IAR) for Dutch (air) ports?**

	1 Strongly disagree	2 Disagree	3 Neutral	4 Agree	5 Strongly agree
i. The IAR allowed us to identify challenges and problems we encountered during the COVID-19 response at / in (air) ports.					
ii. The IAR enabled participants to share experiences and solutions during the COVID-19 response in / at (air) ports.					
iii. The IAR contributed to improved collaboration among various public health partners and disciplines involved during the COVID-19 response.					
iv. The IAR contributed to improved multi-sectoral cooperation and coordination among parties involved in the COVID-19 response in / at (air) ports					
v. During the IAR, there was room for participants to make suggestions on how to improve the COVID-19 response in / at (air) ports.					

2. **To what extent do you agree with the following statements about the in(tra)-action review (IAR) method?**

	1 Strongly disagree	2 Disagree	3 Neutral	4 Agree	5 Strongly agree
i. The presentation on the method and process of the meeting was clear and useful.					
ii. **The introduction** outlining the Dutch situation and presenting a timeline of key events and actions was useful and efficient.					
iii. **Session 1**, in which we discussed the implementation of various mitigation measures at airports/ports, was efficient.					
iv. **Session 2**, in which we discussed cooperation in COVID-19 combat at airports/ports, was efficient.					
v. The number of participants during the meeting and its various components was adequate.					

vi. The participants had the right profile to participate in the IAR.					
vii. The methods used in this IAR could also be effective for an evaluation of other subjects or other events.					
x. In general, I consider the IAR methodology to be effective in delivering objective and concrete results.					

3. To what extent do you think the results of the IAR can contribute to:

	1<br>Not at all	2<br>Hardly	3<br>Neutral	4<br>Pretty sure	5<br>Totally sure
i. Timely agenda setting of key deficiencies in COVID-19 response at airports/ports.					
ii. Putting deficiencies in coordination and cooperation on the agenda.					
iii. Identifying, replicating and retaining solutions and efficient practices.					
iv. Support and strengthen individuals to better meet the challenges of the response.					
v. Bringing attention to solutions or new capacity developed during the COVID-19 response.					

4. Do you have any other comments that relate to the IAR method?

5. Do you have any other comments that relate to the results and outcomes of the IAR?

