# Supplementary material for: Evaluating intra-action reviews at points of entry: ongoing learning opportunities during the COVID-19 pandemic
Source: BMC Public Health. 2023 Jan 6;23:36. doi: 10.1186/s12889-022-14706-4 (PMC9816518; doi:10.1186/s12889-022-14706-4)
Supplement: Supplementary file 5 — Additional file 5. [file 12889_2022_14706_MOESM5_ESM.pdf]

Additional file 5 – Follow up of actions questionnaire. PDF file. *[originally sent in Dutch]*

Follow up of actions for ports

Actiepunten	Ondernomen actie
1. Regional agreements are made on how ships make a COVID-19 notification to the GGD if they are already in the port.	o Completed<br>o Partially completed<br>o Not completed<br><br>Explanation:
2. GGDs will evaluate internally how confidential medical information is handled and bring this issue to the attention of agencies.	o Completed<br>o Partially completed<br>o Not completed<br><br>Explanation:
3. A three-weekly consultation will be set up with the Ministry of Health, the NIPH, doctors of infectious disease control of the PHSs and representatives of ports and safety regions. Each PHS will supply contact information to the NIPH for the establishment of this group.	o Completed<br>o Partially completed<br>o Not completed<br><br>Explanation:
4. PHS designate knowledge holders for port-related issues.	o Completed<br>o Partially completed<br>o Not completed<br><br>Explanation:
5. The NIPH and the PHS remain in regular and good contact and intensify this contact where possible.	o Completed<br>o Partially completed<br>o Not completed<br><br>Explanation:
6. In the previously mentioned consultation to be established with the Ministry of Health, the NIPH and the PHSs, the dossiers cruise shipping and quarantine locations will be discussed.	o Completed<br>o Partially completed<br>o Not completed<br><br>Explanation:

Follow up of actions for airports

Dear Participant,

You hereby receive the final version of the summary report of the In(tra)-Action Review (IAR) airports in which you participated. A number of action points emerged from the IAR. We would like to hear from you on which action points from the report you have already taken action (and if so, which action). You can fill this in in the table below.

We would like to receive your response in this table by [date].
We will process your input and aim to complete the evaluation next week.

Kind regards,

On behalf of the IAR organizing team,

[Names]

Action item formulated during the IAR discussion for airports and which apply to the regional/local level.	It has already been completed, with the following result:	This action item is ongoing. The following steps have already been taken:	To my knowledge, nothing has been done with it since the IAR, and that's because of:	Other, namely ...:
At the tri-weekly consultation with the Ministry of Health, the aim is to have an infectious disease consultant (MD) participate as part of the PHS. The NIPH can also join by invitation.				
In consultation with the safety regions, it will be examined whether customization is possible at the smaller airports when implementing public health measures.				
The participants agreed to assess internally, within the region/organization, of what cooperation exists and how it can be optimized.				
At Schiphol Airport, arrangements will be made				

for the stranded travelers, thereby including the airlines.				

Are there any needs in which the NIPH can play a role?

Please share here any further comments/remarks or clarifications following the IAR or answering the above questions.

